# Effect of Steel Fiber Content on Shear Behavior of Reinforced Expanded-Shale Lightweight Concrete Beams with Stirrups

**DOI:** 10.3390/ma14051107

**Published:** 2021-02-26

**Authors:** Changyong Li, Minglei Zhao, Xiaoyan Zhang, Jie Li, Xiaoke Li, Mingshuang Zhao

**Affiliations:** 1 International Joint Research Lab for Eco-Building Materials and Engineering of Henan, School of Civil Engineering and Communications, North China University of Water Resources and Electric Power, Zhengzhou 450045, China; zxyanzi@ncwu.edu.cn (X.Z.); lixk@ncwu.edu.cn (X.L.); zhaoms@stu.ncwu.edu.cn (M.Z.); 2 School of Engineering, RMIT University, Melbourne, VIC 3003, Australia; jie.li@rmit.edu.au

**Keywords:** steel fiber reinforced expanded-shale lightweight concrete (SFRELC), reinforced SFRELC beam, stirrups, volume fraction of steel fiber, shear cracking, shear crack width, shear capacity

## Abstract

To determine the validity of steel fiber reinforced expanded-shale lightweight concrete (SFRELC) applied in structures, the shear behavior of SFRELC structural components needs to be understood. In this paper, four-point bending tests were carried out on reinforced SFRELC beams with stirrups and a varying volume fraction of steel fiber from 0.4% to 1.6%. The shear cracking force, shear crack width and distribution pattern, mid-span deflection, and failure modes of test beams were recorded. Results indicate that the shear failure modes of reinforced SFRELC beams with stirrups were modified from brittle to ductile and could be transferred to the flexure mode with the increasing volume fraction of steel fiber. The coupling of steel fibers with stirrups contributed to the shear cracking force and the shear capacity provided by the SFRELC, and it improved the distribution of shear cracks. At the limit loading level of beams in building structures at serviceability, the maximum width of shear cracks could be controlled within 0.3 mm and 0.2 mm with the volume fraction of steel fiber increased from 0.4% to 0.8%. Finally, the formulas are proposed for the prediction of shear-cracking force, shear crack width, and shear capacity of reinforced SFRELC beams with stirrups.

## 1. Introduction

In the construction of high-rise buildings and large span spatial structures as well as long span bridges, the self-weight of the structure is a key issue which should be of concern. This promotes the development of lightweight aggregate concrete. Due to the different resources and manufactured methods, many kinds of lightweight aggregates are market supplied. The material widely used for structural concrete in China is the sintered expanded-shales. After a series experimental studies, a high-performance steel fiber reinforced expanded-shale lightweight concrete (SFRELC) is produced. The sintered expanded-shale and the ceramsite sand are used for the aggregates of SFRELC to realize the lightweight itself. On the previous studies, SFRELC has the characteristics of structural material with desirable mechanical properties [1,2], volume stability [3], and good durability [4]. Moreover, the bond of a steel bar with SFRELC is reliable [5]. This provides the foundation of SFRELC applied in structural engineering.

The studies on the flexural behavior of reinforced SFRELC beams under static concentrated loads indicated that the cracking moment, flexural stiffness, and bearing capacity as well as ductility are effectively promoted with the increasing volume fraction of steel fibers from 0.4% to 1.6%, and the crack growth is confined with decreased spacing and width [6,7,8]. A fatigue life of reinforced SFRELC superposed beams with ductile failure mode was prolonged by preventing fatigue fracture of longitudinal tensile rebar and reducing crack growth, and the failure could be expected with the trend curves of fatigue flexural stiffness [9]. By the experimental study on shear-cracking force and shear crack extension, mid-span deflection, shear failure mode, and shear capacity, the effective enhancement of steel fibers was confirmed on the shear behavior of reinforced SFRELC beams without stirrups; however, the brittle failure could not be prevented with the rupture of expanded shales along diagonal cracks [10]. This indicates the efficiency of steel fiber to increase the behavior of reinforced SFRELC beams in tension.

Currently, few studies were reported on the shear behavior of steel fiber reinforced lightweight aggregate concrete (SFRLAC) beams with stirrups. Nes and Øverli [11] studied the structural behaviors of layered beams with SFRLAC and conventional concrete, and the results show that the stirrups might be reduced due to the ductility of SFRLAC in tension increased by steel fibers; the shear capacity of test beams increased with the increased content of steel fiber and depended on the distribution and orientation of steel fibers. Jiao et al. [12] investigated the shear behavior of reinforced SFRLAC beams, and the results indicate that accompanied with the rupture of coarse lightweight aggregate on the failure sections, the failure of test beams changed from shear to flexural shear with the increasing amount of steel fiber; the shear capacity provided by stirrups and the shear ductility of test beams could be increased by the presence of steel fiber due to the enhanced bond property of stirrups with SFRLAC.

In view of the above research studies, the interaction of steel fiber with stirrups needs to be identified. However, there is lack of research on the shear behavior of reinforced SFRLAC beams, let alone the shear behavior of reinforced SFRELC beams. In this condition, we may learn from the shear functions of steel fiber and stirrups in steel fiber reinforced concrete (SFRC) beams based on the similar shear mechanisms. A number of experimental studies indicate that steel fibers have potential advantages when used to supplement or replace stirrups in reinforced SFRC beams, which can effectively improve the shear behavior in combination with stirrups. Zhao et al. [13,14,15] indicate that the improvement of steel fibers on shear-cracking force and shear capacity of reinforced SFRC beams is consistent to that of the tensile strength of SFRC, which was independent of the stirrups. Meanwhile, the distribution of diagonal cracks was affected by the interaction of steel fibers and stirrups. Stirrups in a small spacing made the uniform width of diagonal cracks, while steel fibers limited the extension of these cracks. Researchers exhibit that [16,17,18,19] the inclusion of steel fibers in adequate content can change the failure mechanism from brittle shear into ductile flexure. This confirms the possibility of using steel fiber reinforcement instead of increasing the stirrups amount to achieve analogous performance for reinforced SFRC beams. Similarly, steel fiber reinforcement can be used with a combination of reduced stirrups to satisfy the shear capacity and ductility of reinforced SFRC beams [19,20,21,22,23]. As a result, replacing minimum stirrups was feasible by a certain amount of steel fibers in the condition of reinforced SFRC beams in flexural failure [24,25,26,27]. On the other hand, the minimum stirrups play a better role of controlling shear cracks than steel fibers in terms of more diagonal cracks with small width at failure, and they effectively mitigate the size effect of depth on the shear capacity of reinforced SFRC beams [28,29]. A study performed by di Prisco and Romero [30] shows that in combination with stirrups, the hybrid fibers are quite substantial to the diagonal shear of thin-webbed reinforced concrete beams. Moreover, a certain amount of steel fiber trends are more effective than stirrups for the control of shear cracks on reinforced SFRC beams with different shear span to depth ratios, while the contribution of steel fibers on the shear capacity of reinforced SFRC beams depends on the situations of different ratios of stirrups [31,32,33,34].

## 2. Research Significance

Due to the shear behavior of reinforced concrete beams closely connected to the properties of concrete, the attention should be noted on the shear behavior related to aggregate type, which affects the strength of concrete, the dowel action of longitudinal reinforcement, the interlocking action of aggregates along the cracked section, and the bond–slip of stirrups with concrete [5,10,35,36,37]. Obviously, current study is insufficient on the shear behavior of reinforced SFRELC beams, and it cannot be applied as a reliable design consideration. In this paper, an experimental research study was carried out to fill in the research gap on the shear behavior of reinforced SFRELC beams with stirrups. Ten test beams were designed to pronounced shear failure. Test results are directly compared to the previous study of reinforced SFRELC beams without stirrups [10] and evaluated with the specifications of current design codes [37,38,39]. Finally, the predictive formulas for the shear-cracking force, shear capacity, and shear crack width of reinforced SFRELC beams are proposed considering the featured strengthening effect of steel fibers with stirrups.

## 3. Experimental Work

### 3.1. Production of SFRELC

Grade 52.5 ordinary silicate cement, sintering expanded shale, ceramsite sand, polycarboxylic acid super-plasticizer, milling steel fiber, and tap water were used for SFRELC. The properties of cement were 27.4% water requirement of normal consistency, 51.7 MPa of compressive strength, and 9.2 MPa of tensile strength at 28 days. The sintered expanded shale was continuous grading with a maximum particle size of 20 mm, bulk and particle densities of 800 and 1274 kg/m^3^, cylinder compressive strength of 5.0 MPa, and water absorption for 1 h of 6.1%. The ceramsite sand was the by-product of sintered expanded shale in continuous grading with particle size of 1.6–5 mm, a fineness modulus of 3.56, bulk and particle densities of 946 and 1659 kg/m^3^, and water absorption for 1 h of 9.0%. The steel fiber was of milling type in length l_f_ = 36 mm and equivalent diameter d_f_ = 1.35 mm, the aspect ratio l_f_/d_f_ = 27.1. The mix water was the tap water of Zhengzhou city, China.

The mix proportion of SFRELC was primarily designed by the absolute volume method and adjusted to keep the slump of fresh SFRELC at (120 ± 20) mm [7,10,37,40]. The volume fraction v_f_ of steel fiber was changed to 0%, 0.4%, 0.8%, 1.2%, and 1.6%. The fiber factor λ_f_ = 27.1v_f_. The additional water for pre-wetting the sintered expanded-shale and ceramic sand was computed with 1 h water absorptions.

The fresh SFRELC was mixed by a single-horizontal-shaft mixer. The expanded shale and ceramic sand with the additional water was firstly pre-wetted in the mixer for 1 h. Then, the cement and half dosage of the mix water were added and mixed for 30 s. The super-plasticizer and the residual mix water were added during the mixing, and the steel fiber was sprinkled into the mixer and mixed for 3 min [3,4,5,6,7,10].

According to the specification of China code JG/T472 [40], the standard specimens for the test of strength of SFRELC were fabricated and cured at the same condition of test beams. The splitting tensile strength f_ft_ of SFRELC was tested by three cubes of 150 mm, and the axial compressive strength f_fc_ was tested by three prisms of 150 mm × 150 mm × 300 mm. The tested values are presented in Table 1.

### 3.2. Design and Fabrication of Test Beams

The reinforced SFRELC beams were designed to have a pronounced shear behavior with the popular rectangular section. As found in previous study [10], the shear capacity of reinforced SFRELC beams without web reinforcements depends on the shear span to depth ratio, the longitudinal reinforcement ratio, and the tensile strength of SFRELC. The effects of steel fiber focused on the bearing capacity of SFRELC in the shear and compression zone, the interlock among aggregates along sides of diagonal cracks, and the dowel action of longitudinal tensile rebar. In the designing of test beams with stirrups, the shear capacity V_u_ was supposed as the independent contribution of stirrups with the composition of the beams without web reinforcements [37,38,39], that is,

(1) $$V_{u}=V_{\mathrm{fcu}}+V_{\mathrm{sv}}.$$

The shear resistance V_fcu_ provided by SFRELC was computed by formula [10],

(2) $$V_{\mathrm{fcu}}=\frac{0.115+0.192\lambda +28.7\rho }{\lambda -0.6}f_{\mathrm{ft}}{\mathrm{bh}}_{0}.$$

The shear resistance V_s_ provided by stirrups was computed by formula [38,39],

(3) $$V_{\mathrm{sv}}=\rho_{\mathrm{sv}}f_{\mathrm{yv}}{\mathrm{bh}}_{0}.$$

In the formulas, b is the sectional width; h_0_ is the sectional effective depth, h_0_ = h − c_s_ − d/2, h is the sectional depth, c_s_ is the concrete cover; f_ft_ is the tensile strength of SFRELC; ρ is the ratio of longitudinal rebars, ρ = A_s_/bh_0_, taken ρ = 3.0% when ρ ≥ 3.0%; A_s_ is the cross-sectional area of longitudinal tensile rebars; λ is the shear span to depth ratio, λ = a/h_0_, taken λ = 4 when λ ≥ 4, a is the shear span; ρ_sv_ is the ratio of stirrups, ρ_sv_ = A_sv_/bs; A_sv_ is the cross-sectional area of stirrups; s is the spacing of stirrups; f_yv_ is the yield strength of stirrups.

Meanwhile, the flexural capacity of test beams was designed by using the formulas provided in previous study [7],

(4) $$M_{\mathrm{fu}}=f_{y}A_{s}\left(h_{0}-\frac{x}{2}\right)+f_{\mathrm{ftu}}{\mathrm{bx}}_{t}\left(h-\frac{x}{2}-\frac{x_{t}}{2}\right)$$

(5) $$f_{\mathrm{fc}}\mathrm{bx}=f_{y}A_{s}+f_{\mathrm{ftu}}{\mathrm{bx}}_{t}$$

(6) $$x_{t}=h-\frac{x}{0.75}$$

(7) $$f_{\mathrm{ftu}}={1.86\lambda }_{f}f_{t}$$

where M_fu_ is the moment capacity of the reinforced SFRELC beam; x is the compressive depth of the cross-section; x_t_ is the equal tensile depth of the cross-section; f_y_ is the yield strength of longitudinal tensile rebars; f_ftu_ is the equal tensile strength of SFRELC in the tensile zone of the cross-section, f_t_ is the tensile strength of the SFRELC matrix; and λ_f_ is the fiber factor, i.e., the product of v_f_ and l_f_/d_f_.

**Figure 1:**
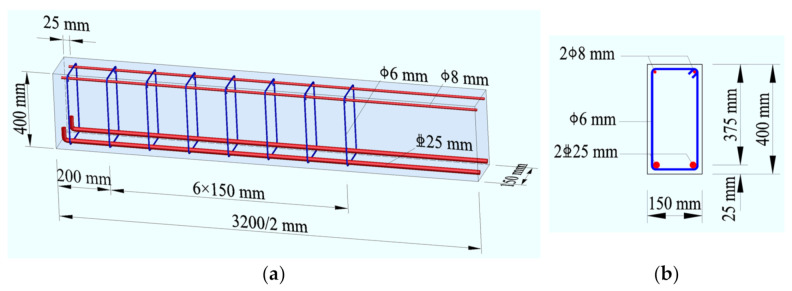
Layout of reinforcements in test beams: (**a**) longitudinal section; (**b**) transversal section.

Based on above formulas, ten beams were designed; two of them identified as a/b were the same in each group, as presented in Table 1. The width b = 150 mm, the depth h = 400 mm, the length l = 3200 mm, and the span l_0_ = 2900 mm. Details of the rebars in the test beams are presented in Figure 1. The longitudinal tensile rebars were two hot-rolled HRB500 ribbed rebars with diameter d = 25 mm, the concrete cover c_s_ = 25 mm. The constructional compression rebars were two hot-rolled HPB300 plain rebars with a diameter of 8 mm. The stirrups were the hot-rolled HPB300 plain rebar with a diameter of 6 mm.

The tensile strength of rebars was measured. The yield strength f_y_ = 517 MPa and the ultimate strength was 598 MPa for the hot-rolled HRB500 ribbed rebar with a diameter of 25 mm. The yield strength f_yv_ = 398 MPa and the ultimate strength was 529 MPa for the hot-rolled HPB300 plain rebar with a diameter of 6mm.

The shear span to depth ratio of the test beams λ = 2.5, the shear span of test beam a = 905 mm. The spacing of stirrups s = 150 mm, the stirrups ratio ρ_sv_ = 0.25%. The measured sectional dimension, the shear force V_u(1)_ computed by Formula (1), and that V_u(4)_ (= M_fu_/a) computed by Formula (4) for the test beams are also presented in Table 1. This shows that the test beams were designed to fail in shear due to V_u(1)_ being less than V_u(4)_.

The fresh SFRELC was cast twice into the steel form, and it was compacted with the outside vibrators fixed on the steel form. The screeding top-surface was covered by plastic film for 48 h before being demolded. After being demolded, the test beams were cured with sprayed water for 7 days and then placed in the outdoor environment before testing.

### 3.3. Test Method

**Figure 2:**
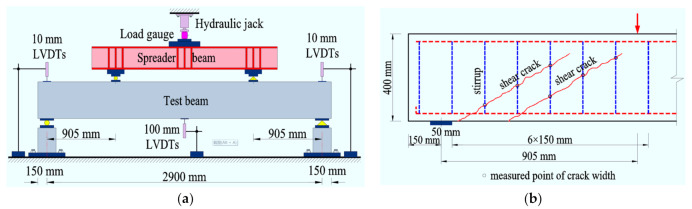
Test method: (**a**) testing apparatus; (**b**) measuring of shear crack width.

As presented in Figure 2, the four-point bending test was carried out with the shear span a = 905 mm and the pure bending segment of 1090 mm. The beam was simply supported on the supports. A hydraulic jack fixed on a loading frame were used to exert the symmetrical concentrated loads on the top surface of the test beam by a spreader steel beam.

Load gauges were linked to the hydraulic jack to measure the values of loads. The mid-span deflection was determined by three displacement sensors (LVDTs-linear variable differential transformer) placed at the supports and mid-span section of the test beam. The width of the shear crack on the side surfaces that intersected the stirrups of the test beam was detected with a reading microscope. The angle of the shear crack with the bottom surface of the test beam was determined as the diagonal angle of the shear crack.

## 4. Test Results and Analyses

### 4.1. Patterns of Crack Distribution and Failure

**Figure 3:**
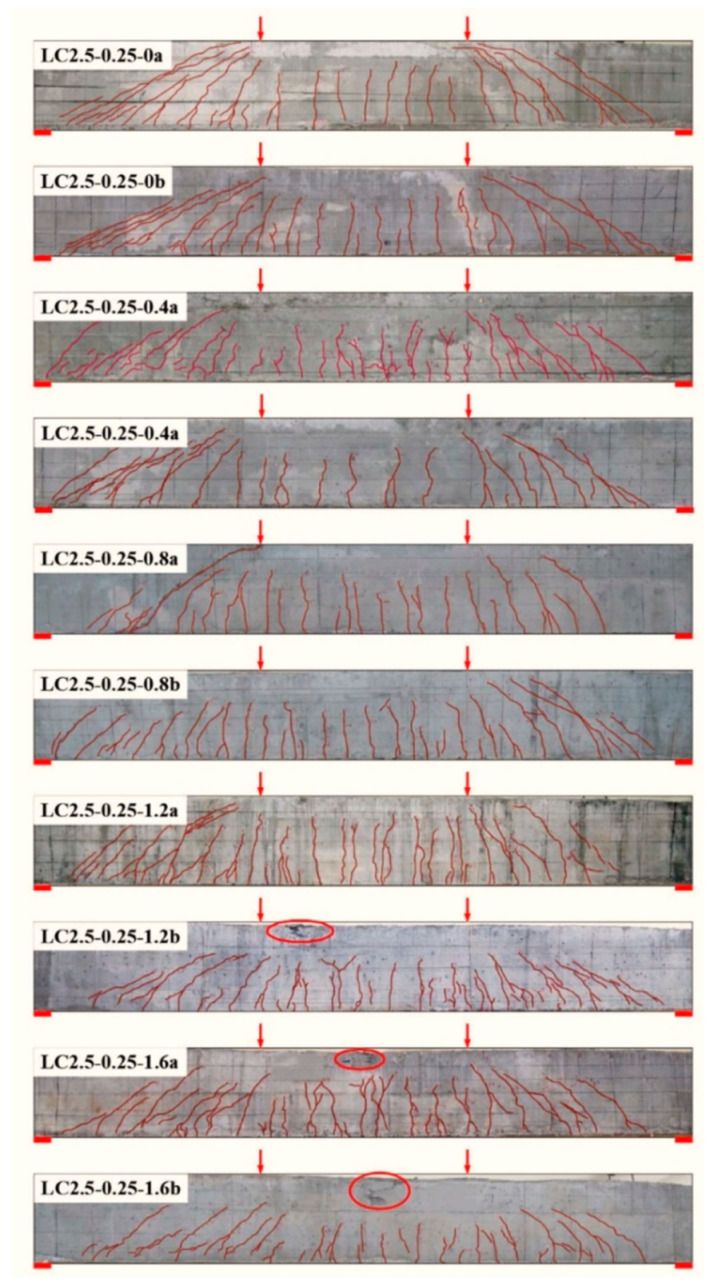
Crack distribution and failure mode of test beams.

The crack distribution and failure mode of test beams are presented in Figure 3. The shear cracks appeared on the shear span after the flexural cracks appeared at the pure bending segment. This indicates that the shear-cracking resistance of the diagonal section was higher than the flexural cracking resistance of the normal section for test beams. 

For test beams LC2.5-0.25-0a/b without steel fiber reinforcement, one or two main diagonal cracks appeared on the shear span, and the shear failure with wider diagonal cracks took place. With the increasing volume fraction of steel fibers v_f_, the number of shear cracks increased, the diagonal cracks slowly extended, and the diagonal angle of the main shear crack tended to be reduced slightly. Meanwhile, the shear-compression zone existed clearly with uncrack depth near loading points. The shear failure with smaller diagonal cracks took place on test beams with v_f_ ≤ 0.8%, and the failure in the shear compression mode took place on test beam LC2.5-0.25-1.2a with v_f_ = 1.2%. This is in keeping with the pronounced shear failure of design. 

However, test beam LC2.5-0.25-1.2b with v_f_ = 1.2% failed in shear accompanied by flexure, and test beams LC2.5-0.25-1.2a/b with v_f_ = 1.6% failed in flexure. Due to the shorter diagonal cracks extended on shear span, a larger shear-compression zone was reserved near the loading points. This effectively increased the shear resistance provided by the uncracked SFRELC in shear compression. Meanwhile, the presence of stirrups confined the extension of diagonal cracks, which benefits the steel fibers bridging the diagonal cracks to transfer tensile stress. Hence, the enhancement of steel fibers on the shear capacity of reinforced SFRELC beams with stirrups was higher than that of reinforced SFRELC beams without stirrups. This led to a lower value predicted by Formula (2), which was based on the research of reinforced SFRELC beams without stirrups [10]. In this condition, the positive interaction of steel fibers and stirrups on the shear capacity of reinforced SFRELC beams should be considered.

**Figure 4:**
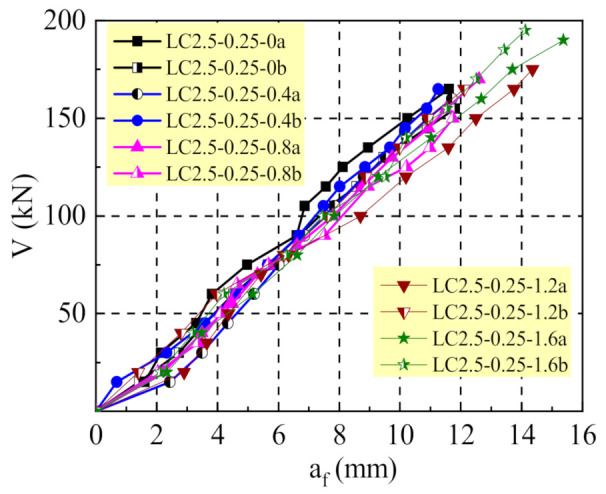
Mid-span deflection of test beams.

As exhibited in Figure 4, similar changes existed for the mid-span deflection of test beams. This indicates a slight difference on the dowel action of longitudinal reinforcement. Therefore, the function of steel fibers was reflected to increase the number of shear cracks and to decrease the crack width. This enhances the interlocks of aggregates along the cracked section, and it reduces the bond–slip of stirrups with SFRELC.

**Figure 5:**
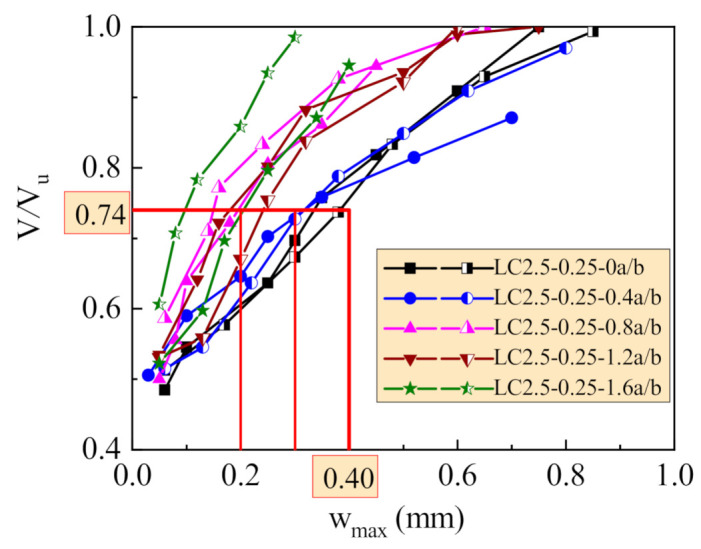
Shear crack widths of test beams at different loading levels.

Figure 5 presents the maximum width of the main shear cracks on test beams at different loading levels V/V_u_. With the increase of v_f_, the shear cracking took place at an increased loading level, and the crack opened slowly with a smaller width at the same loading level after cracking. At the limit loading level of 0.74 for beams of building structures at serviceability, the maximum width of shear cracks on beams without steel fiber was close to 0.4 mm, which is controlled at normal environment; when the v_f_ = 0.4%, the maximum width reduced to be within 0.3 mm; when the v_f_ ≥ 0.8%, the maximum width reduced to be within 0.2 mm. This provides a good adaptability of reinforced SFRELC beams in a more severe environment [38,39].

### 4.2. Shear-Cracking Force

The shear-cracking force V_cr_ computed by the loads corresponding to shear cracking are presented in Table 2. In this study, the shear cracking was determined as an initial inclined web crack or a shear–flexural crack was beginning to turn to the load point [10,37,38,39]. With the increase of v_f_, the initial shear crack width tended to reduce from 0.06 mm to 0.03 mm. This exhibited that steel fibers played a role to confine the opening of shear cracks.

To facilitate compressive analysis, the shear stress τ_cr_ was computed as the shear-cracking force on the unit sectional area,

(8) $$\tau_{\mathrm{cr}}=V_{\mathrm{cr}}/{\mathrm{bh}}_{0}.$$

**Figure 6:**
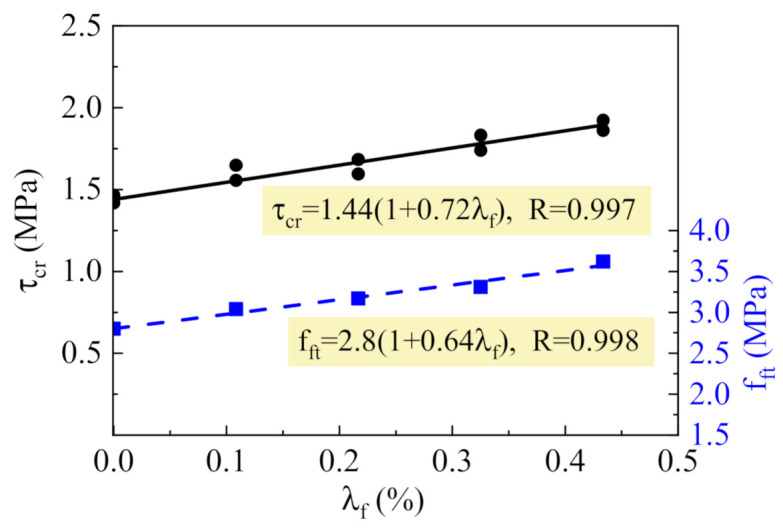
Changes of τ_cr_ and f_ft_ with λ_f_.

As presented in Figure 6, the shear stress τ_cr_ and the tensile strength f_ft_ have linear relationships with the fiber factor λ_f_. The digits 0.72 and 0.64 before λ_f_ in the fitness formula are the strengthening coefficients of steel fiber on τ_cr_ and f_ft_, respectively. 

Obviously, the strengthening coefficient 0.72 of τ_cr_ is greater than the 0.64 of f_ft_. This is differing from the fact that the τ_cr_ of reinforced SFRELC beams without stirrups increased almost the same with f_ft_ [10]. Therefore, the reinforcement of steel fiber on the shear cracking resistance is not only controlled by the tensile strength of SFRELC but also improved by the stirrups due to the beneficial stress distribution of SFRELC at shear span.

To simplify the computation, the beneficial effect of stirrups on shear cracking resistance can be considered as a reliability reserve. The same formula can be used for predicting the shear-cracking force of reinforced SFRELC beams with or without web reinforcement [10],

(9) $$V_{\mathrm{cr}}=\left(\frac{2.45}{\lambda +3.5}+\frac{20\rho }{\lambda +1.1}\right)f_{\mathrm{ft}}{\mathrm{bh}}_{0}.$$

Table 2 presents the calculated values of V_cr_ by using Formula (9). Based on the statistical analysis, the average of ratios of test to calculation results is 1.034 with the coefficient of variation as 0.027. Therefore, a slightly lower value is predicted by using Formula (9).

### 4.3. Shear Capacity

The test peak shear force V_u_ computed by the peak load on test beams is summarized in Table 2. Due to the failed shear and shear-flexural patterns of test beams with v_f_ = 1.2%, the peak shear forces of them were falling in between shear failure and flexural failure. Due to failed flexural pattern of test beams with v_f_ = 1.6%, the peak shear forces were lower than the shear capacity.

**Figure 7:**
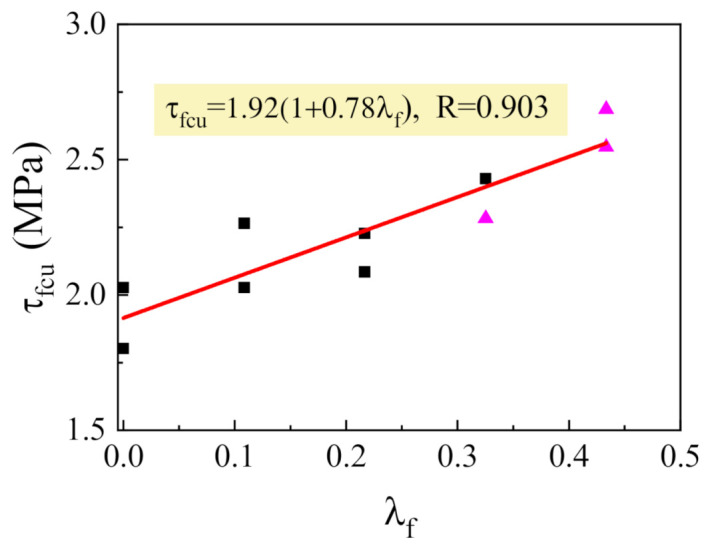
Changes of τ_fcu_ with λ_f_.

In the premise of the shear force provided by stirrups calculated by Formula (3), the beneficial effects of stirrups can be comprehensively counted by correcting the shear force subjected by SFRELC. From Formula (1), we can obtain V_fcu_ = V_u_ − V_sv_. Referenced to Formula (8), let τ_fcu_ = V_fcu_/bh_0_. The fitted linear relationship of τ_fcu_ with λ_f_ is presented in Figure 7. The strengthening coefficient of steel fiber that interacted with stirrups on shear resistance provided by SFRELC is 0.78, which is greater than that of 0.64 (see Figure 6) for tensile strength of SFRELC. This reflects the coupling effect of steel fibers with stirrups on the shear capacity provided by SFRELC.

Referencing the form f_ft_ = f_t_(1 + 0.64λ_f_), Formula (2) can be transferred to be Formula (10) as follows,

(10) $$V_{\mathrm{fcu}}=\frac{0.115+0.192\lambda +28.7\rho }{\lambda -0.6}f_{t}\left(1+0.78\lambda_{f}\right){\mathrm{bh}}_{0}.$$

Referring to the design principle of shear capacity with sufficient reliability for reinforced concrete and SFRC beams specified in worldwide design codes such as CSA 23.3-04 [41], Eurocode 2 [42], ACI 318-14 [43], JGJ/T465 [39], and ACI 544.4R [44], a lower computed shear capacity of reinforced SFRELC beams can be calculated on the basis of the design formula of lightweight-aggregate concrete beams [37],

(11) $$V_{u}=\frac{1.5\beta_{\rho }}{\lambda +1.0}f_{t}\left(1+0.78\lambda_{f}\right){\mathrm{bh}}_{0}+\frac{A_{\mathrm{sv}}f_{\mathrm{yv}}}{s}h_{0}$$

where λ = 1 when λ < 1, and λ = 4.0 when λ > 4.0. β_ρ_ = 0.7 + 20ρ.

The computed shear capacities of test beams are presented in Table 2. By using Formula (11), the shear capacities of test beams are predicted with the average reduction of 30.8%. This gives a conservative shear capacity of the reinforced SFRELC beams with stirrups.

### 4.4. Shear-Crack Width

To predict the shear-crack width of reinforced SFRELC beams in normal service condition, the formulas for reinforced SFRC beams are revised and expressed as follows [14],

(12) $$w_{\max}=\left(1-1.21\lambda_{f}\right)\frac{\sigma_{\mathrm{sv},m}}{E_{\mathrm{sv}}}h_{0}\cdot \sqrt{0.83-0.64{\lambda h}_{0}/l_{0}}$$

(13) $$\sigma_{\mathrm{sv},m}=\frac{V-0.9V_{\mathrm{cr}}}{0.6{\lambda \rho }_{\mathrm{sv}}{\mathrm{bh}}_{0}}$$

where w_max_ is the maximum width of shear cracks intersected with stirrups; σ_sv,m_ is the tensile stress of stirrups; E_sv_ is the modulus of elasticity of stirrups; and l_0_ is the span.

**Figure 8:**
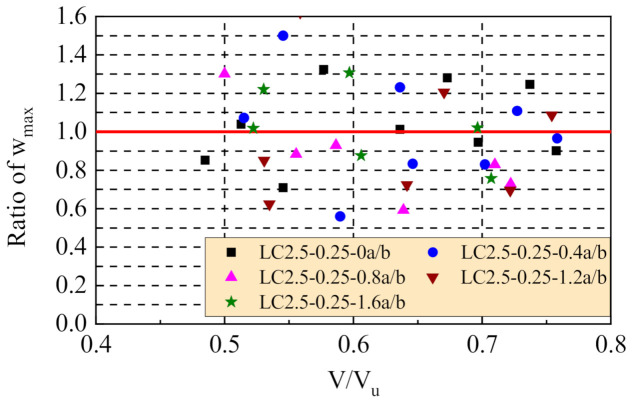
Comparison of test to calculation results of the maximum diagonal crack width.

Within the loading level of the normal service state, the comparison of the test and computation results of the maximum width of shear cracks is presented in Figure 8. The ratios varied from 0.346 to 1.621, the variation tended to decrease with the increasing volume fraction of steel fiber. Based on the statistical analysis, the ratio was 1.0 on average with a coefficient of variation of 0.274.

Due to the randomness of shear crack extension, the width of the main diagonal cracks was affected by complex factors. It is difficult to accurately predict the width of shear cracks. Therefore, the width of shear cracks is always indirectly limited by using the design method of a conservative shear capacity [37,38,39]. In this study, the design shear capacity of test beams by Equation (11) was reduced by 30.8% compared with the test results. This indicates the real loading level at normal serviceability will be reduced by 30.8% from the limit loading level of 0.74; that is, 0.74/1.308 = 0.566. From Figure 5, the crack width could be strictly controlled within 0.2 mm even 0.1 mm. This provides a good condition of reinforced SFRELC beams applied in severe environment.

## 5. Conclusions

Based on the shear testing of reinforced SFRELC beams with stirrups, and compared with the shear behavior of reinforced SFRELC beams without stirrups in previous study, conclusions are drawn as follows:

(1) Steel fiber had a higher strengthening effect coupling with stirrups on the shear-cracking resistance of reinforced SFRELC beams. For simplifying the calculation, the beneficial effect can be neglected to provide a conservative result only considering the strengthening of steel fiber on the tensile strength of SFRELC.
(2) With the increase of steel fiber content, the shear failure could be modified with good ductility and transferred to the flexure. The stirrups improved the distribution of shear cracks with reduced spacing and width. This provided a good condition of SFRELC subjected to shear force to improve the shear capacity provided by SFRELC. Considering the effect of steel fiber reinforcement on the shear capacity of beams provided by SFRELC, the formulas are proposed for the prediction and the design of shear capacity of reinforced SFRELC beams with stirrups.
(3) With the increase of steel fiber content, the maximum width of shear cracks became smaller after shear cracking, and it extended slowly at the same loading level of test beams. The reduction of maximum width of shear cracks promoted the adaptability of reinforced SFRELC beams in a severe environment. Based on the test results, the formula for the prediction of maximum width of shear cracks is proposed.
(4) The study was limited to the changes of steel fiber content in condition of the constant shear span to depth ratio and stirrups ratio. To build a broad recognized method for the design of reinforced SFRELC beams under shear stress, many more systematical research studies need to carried out to identify the effects of multiple factors.

## Figures and Tables

**Table 1:** Dimension, influencing factor of test beams, and main tested results.

Identifier	b (mm)	h_0_ (mm)	vf (%)	Strength of SFRELC (MPa)		Computed Shear Force (kN)	
				ffc	fft	V_u(1)_	V_u(4)_
LC2.5-0.25-0a	150	362	0	36.4	2.80	143	177
LC2.5-0.25-0b	155	362	0	36.4	2.80	145	178
LC2.5-0.25-0.4a	150	362	0.4	42.4	3.04	151	179
LC2.5-0.25-0.4b	150	362	0.4	42.4	3.04	151	179
LC2.5-0.25-0.8a	155	362	0.8	49.2	3.17	157	182
LC2.5-0.25-0.8b	155	362	0.8	49.2	3.17	157	182
LC2.5-0.25-1.2a	150	362	1.2	51.6	3.31	160	182
LC2.5-0.25-1.2b	150	362	1.2	51.6	3.31	160	182
LC2.5-0.25-1.6a	150	362	1.6	52.3	3.62	170	182
LC2.5-0.25-1.6b	155	364	1.6	52.3	3.62	171	182

**Table 2:** Test and calculation results of shear-cracking force, shear capacity, and failure mode.

Identifier	V_cr_ (kN)		Failure Pattern	V_u_ (kN)		
	Test	Computed		Test	Computed by Formulas (1), (3) and (10)	Computed by Formula (11)
LC2.5-0.25-0a	80	77.7	Shear	165	143	124
LC2.5-0.25-0b	80	79.8	Shear	156	145	125
LC2.5-0.25-0.4a	90	84.3	Shear	178	151	130
LC2.5-0.25-0.4b	85	84.3	Shear	165	151	130
LC2.5-0.25-0.8a	90	91.8	Shear	180	160	137
LC2.5-0.25-0.8b	95	91.8	Shear	172	160	137
LC2.5-0.25-1.2a	100	90.3	Shear	187	166	141
LC2.5-0.25-1.2b	95	90.3	Shear-flexural	179	166	141
LC2.5-0.25-1.6a	105	100.4	Flexural	201	173	147
LC2.5-0.25-1.6b	105	103.1	Flexural	198	175	149

## Data Availability

Data is contained within the article.
